# Association between COVID-19 and outstanding academic performance at a Spanish university

**DOI:** 10.1186/s13690-023-01225-w

**Published:** 2023-12-13

**Authors:** Fares Amer, Tamara López, Mario Gil-Conesa, Silvia Carlos, Arturo H Ariño, Francisco Carmona-Torre, Miguel A. Martínez-González, Alejandro Fernandez-Montero

**Affiliations:** 1 Department of Preventive Medicine and Public Health, University of Navarra, Pamplona, Navarra Spain; 2 Navarra Institute for Health Research (IdiSNA), Pamplona, Navarra Spain; 3 Department of Environmental Biology, University of Navarra, Pamplona, Navarra Spain; 4 Institute for Data Science and Artificial Intelligence (DATAI), University of Navarra, Pamplona, Navarra Spain; 5 Biomedical Research Network Centre for Pathophysiology of Obesity and Nutrition (CIBEROBN), Carlos III Health Institute, Madrid, Spain; 6 Department of Nutrition, Harvard T.H Chan School, Boston, MA USA; 7 Department of Occupational Medicine, University of Navarra Clinic, Av. Pio XII, 36. 31008, Pamplona, Navarra Spain; 8 COVID-19 Department, University Clinic of Navarra, Pamplona, Navarra Spain; 9 Infectious Diseases Service, University Clinic of Navarra, Pamplona, Navarra Spain

**Keywords:** COVID-19, SARS-CoV-2, Long COVID, Academic performance, Undergraduate students

## Abstract

**Background:**

SARS-CoV-2 is the causative agent of COVID-19 identified in December 2019, an acute infectious respiratory disease that can cause persistent neurological and musculoskeletal symptoms such as headache, fatigue, myalgias difficulty concentrating, among others including acute cerebrovascular disease with a prevalence of 1–35%. The aim of this study is to evaluate the impact of COVID-19 in undergraduate students on their academic performance as an indicator of their intellectual ability and performance in a university that maintained 100% face-to-face teaching during the 2020–2021 academic year.

**Methods:**

A total of 7,039 undergraduate students were analyzed in a prospective cohort study at the University of Navarra. A questionnaire including sociodemographic and behavioral questions was sent. PCRs were performed throughout the academic year for the diagnosis of SARS-CoV-2 infection and students’ academic results were provided by the academic center, adjusted descriptive and multivariate models were performed to assess the association.

**Results:**

A total of 658 (9.3%) participants were diagnosed with COVID-19, almost 4.0% of them achieved outstanding academic results, while uninfected students did so in 7.3%. SARS-CoV-2 infection was associated with a significant decrease in having outstanding academic results (OR = 0.57; 95% CI: 0.38–0.86).

**Conclusion:**

Having COVID-19 disease, decreased academic performance in undergraduate students. Therefore, it is necessary to prevent infection even in the youngest sections of the population.

**Table:** 

**Text box 1. Contributions to the literature**
• COVID-19 has shown to not affect all groups of populations equally, in terms of mortality, where younger population is less affected in that way. But this doesn’t mean they are less susceptible of being harmed in other ways that can affect their daily life and reduce its quality due to cognition impairments.
• This research aims to promote prevention measures in academic centers for a long term to reduce COVID-19 incidence and protect students’ health and academic performance.
• *Some ethnicities are more vulnerable to COVID-19 and more studies should be done to adjust and make prevention measures much more effective.*

## Introduction

SARS-CoV-2 is the causing agent of COVID-19, an acute respiratory disease that causes a variety of symptoms such as fever, headache, cough, pneumonia, breathing difficulty, lung infection, anosmia or body pain [1, 2]. The persistence of some of these symptoms and long-term effects of the disease more than 6 months after surviving to the infection have been described in many articles: severe fatigue (16-69.6%) [2–4], disabling or persisting headache (8–15%) [1, 2, 5, 6], body pain described as myalgia, arthralgia or chest pain (2.4–6.3%) [2]. Moreover, even though the causing agent of COVID-19 is a respiratory virus, an increased risk of having neurological and neuropsychiatric diseases and effects have been described after survival [7–9], including increased incidence of a first psychiatric diagnosis in the following 14 to 90 days [10], PTSD, depression, OCD and panic disorder [11], difficulty in concentrating (5.9–13%) and memory loss (5.6–13%) [2, 12] and insomnia [2, 13]. These persistent symptoms of COVID-19 have been referred to as “Long COVID” [2, 14].

The university period of any student is highly demanding in terms of mental and academic effort, pressure and work. During this period, students need to have good health in order to perform according to their aims and succeed to have access to a promising future. Understandably, any factor that might affect students’ health will impact directly on their academic performance. Specifically, the COVID-19 pandemic has been a big challenge for students to overcome being directly exposed to SARS-CoV-2, where all this context has triggered great distress [15, 16].

Many studies found associations between isolated long COVID symptoms in other diseases and decreased intellectual and work performance, such as headache [17–20], fatigue [21–24], sleep disorders or insomnia [25, 26], and health problems in general [27].

The main objective of this research is to investigate the association of COVID-19 infection effects on students’ academic performance, specifically on the achievement of academic excellence.

## Methods

### Study design

Between August 24, 2020 and May 30, 2021 a cohort study with a 9-month follow-up was conducted among an undergraduate university population at the University of Navarra (Spain) including 8,514 students that were monitored for SARS-CoV-2 infection by PCR testing.

### Participants

**Fig. 1:**
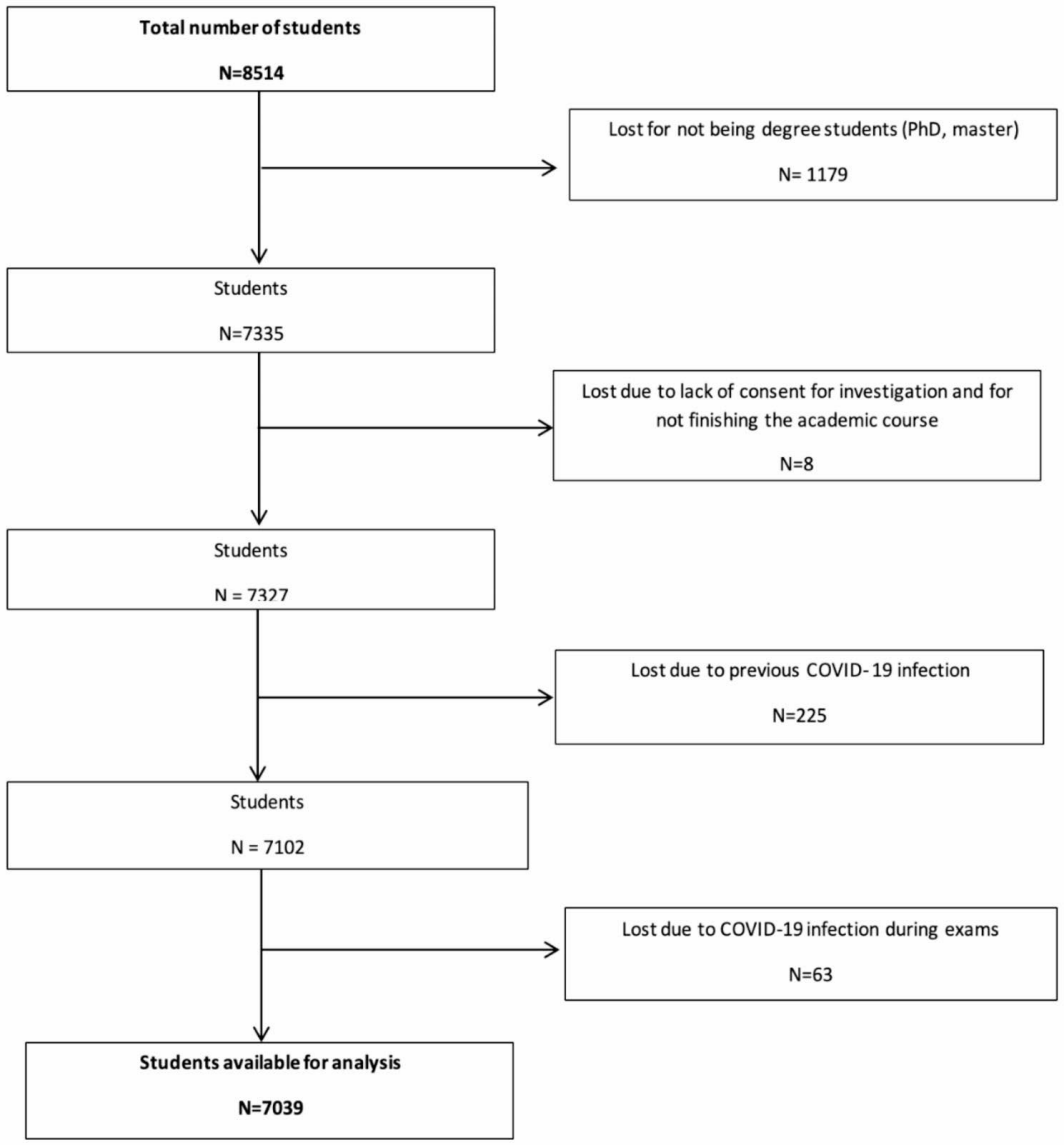
Flowchart of the sample studied at the University of Navarra during the academic year 2020–2021

Participants were undergraduate students from the campuses of Pamplona and Gipuzkoa in the University of Navarra that completed the academic course. Those who completed the questionnaire and gave their consent in the first question were finally included in the study (7,327). Those participants who reported a diagnosis of SARS-CoV-2 infection prior to the start of the course (before August 24, 2020) were excluded, as well as those who didn’t finish the academic year and those who were temporary exchange students or not undergraduate students were also excluded from the analysis (Fig. 1). During the academic year, those students who were diagnosed with COVID-19 15 days before the exams period until the end of the period were excluded also (56 participants).

### Baseline questionnaire and variables

A questionnaire with general questions about COVID-19 symptoms was sent to all students of the university during August and September 2020. This questionnaire included questions on socio-demographics: type of residence during the academic year, number of people with whom they lived, whether or not they lived with children at home, country of origin, hours of daily socialization, living with pets and type of transportation (public transport, walking, car, etc.). They were asked about preventive measures such as handwashing with soap, use of alcohol-based solutions, social distance, gloves, and use of masks (FFP2/3, surgical or cloth mask). They were also asked about data on their weight and height and whether or not they were smokers and about comorbidities (diabetes, cancer, cardiovascular disease, chronic lung disease, impaired coagulation, hypertension, immunosuppression, chronic kidney disease, and chronic liver disease) and any treatment.

In addition to the self-reported variables, objective variables were also collected by the university medical team, such as PCR tests performed on campus, and we obtained from university records the living habitat of the students, country of origin and the academic grades of those who participated.

### COVID-19 diagnosis

Diagnosis of COVID-19 was done with PCR testing following this strategy: (1) Mandatory PCR testing for all students and employees at the beginning of the academic year in August 2020 and after Christmas 2020, (2) PCR testing to symptomatic individuals and close contacts, (3) Weekly random sampling during 8 weeks during the months of October until December, excluding holidays. The back to in-person plan is explained in detail elsewhere [28]. More than 34,000 PCR tests were performed.

### Outcome variable

The main outcome variable was obtaining excellent results, understanding excellence as grades above 9 in the university student population. The score at the University of Navarra is from 0 to 10, with 0 being the worst grade and 10 being the best possible grade. Grades from 9 to 10 are considered as an outstanding result. The other grades categories were “Not passed”, for grades between 0 and < 5, “Passed”, for grades between 5 and < 7, and “Good”, for grades between 7 and < 9. Grades information was provided by the University of Navarra’s central office, at the end of the academic course 2020–2021 after all midterm, final and make-up exams were concluded.

### Statistical analysis

Baseline characteristics of participants are described with frequencies or means and standard deviations, for categorical and continuous variables, respectively. The non-COVID-19 group was considered the reference category. We compared the outstanding academic results using logistic regression models. Obtaining an outstanding result was considered as a dichotomous variable, so an adjusted logistic regression was carried out. Odds Ratios (OR) were estimated and 95% CI were calculated. We built a first model without any adjustment (crude), a second model adjusted for age and sex, and a third multivariable adjusted model considering potential confounders. Finally, stratified analyses and tests for interactions with sex, undergraduate studies, type of housing and origin were performed to ensure the robustness of the results in different scenarios.

Multivariable adjusted models were adjusted for: sex, age, type of housing, BMI, smoker, academic course, hours of socialization, university campus, pets during the academic year and region of origin. Although only 6 variables presented some missing value, the one with the highest number of missing values was hours of socialization with 17.4% of missings, followed by BMI, with 11.3%. A simple imputation was used on the variables when including them as adjustment variables in the multivariate regression.

All p values are two-tailed and a p < 0.05 was considered statistically significant. Stata 12 was used to analyze the data.

This study followed the strengthening of the reporting of observational studies in epidemiology (STROBE) reporting guidelines for cohort studies. The study was conducted in compliance with the study protocol, the current version of the Declaration of Helsinki, and the local, legal and regulatory requirements (Approved by the University of Navarra Ethics committee: 2020.190 on October 30th, 2020).

## Results

Out of 7,039 participants included in our study, all of them were undergraduate students from the campuses of Navarra and Gipuzkoa. A total of 9.3% participants were infected with SARS-CoV-2 during the course, from whom 58% were female, and the mean age was 21.0 SD: 3.21.

Some characteristics show significant differences between students who were infected with SARS-CoV-2 and those who weren’t, the greatest disparity is observed in the type of housing, region of origin, having pets and type of studies. The baseline characteristics of the study participants according to passing COVID-19 or not in students can be seen in Table 1. It is observed that, with respect to the group of students that were not infected, in the infected students group they lived more (76%) at a university apartment or residence during the academic year, were Latin American (20.8%), and were studying humanities degrees (56.53%).

**Table 1:** Baseline characteristics of University of Navarra study participants

COVID-19	Non COVID- 19	COVID-19	p value
Participants, N	6381	658	
Sex, men N, (%)	2530 (39.65%)	278 (42.25%)	0.195
Type of housing N, (%)			< 0.001
“Family home”	2674 (41.91%)	158 (24.01%)	
“University apartment”	2318 (36.33%)	299 (45.44%)	
“University residence or Hall”	1389 (21.77%)	201 (30.55%)	
Pets during the academic year N, (%)			
“No pet”	5475 (85.80%)	606 (92.1%)	< 0.001
“Dog”	639 (10.01%)	38 (5.78%)	
“Cat”	156 (2.44%)	9 (1.37%)	
“Other pets”	111 (1.74%)	5 (0.76%)	
Current smokers N, (%)	652 (10.22%)	69 (10.49%)	0.829
Region of origin N, (%)			< 0.001
“Spain”	5422 (85.12%)	503(76.44%)	
“Latin America”	732 (11.49%)	137(20.82%)	
“Other European countries”	88 (1.38%)	12 (1.82%)	
“Other regions”	128 (2.01%)	6 (0.91%)	
Daily hours of socialization N, (%)			0.044
0	692 (12.51%)	64 (12.24%)	
> 0 to 1	1121 (20.26%)	92 (17.59%)	
> 1 to 2	1486 (26.86%)	155 (29.64%)	
> 2 to 3	1017 (18.38%)	77 (14.72%)	
More than 3	1216 (21.98%)	135 (25.81%)	
Undergraduated studies N, (%)			< 0.001
Science degrees	3601 (56.43%)	286 (43.47%)	
Humanities degrees	2780 (43.57%)	372 (56.53%)	
University campus N, (%)			0.010
Pamplona	5668 (88.83%)	606 (92.10%)	
Gipúzcoa	713 (11.17%)	52 (7.90%)	
Year of study mean, (SD) Age mean, (SD) BMI mean, (SD)	2.8 (1.4) 20.4 (2.1) 21.9 (3.1)	2.6 (1.3) 20.2 (2.1) 22 (3.3)	0.005 0.025 0.502

**Fig. 2:**
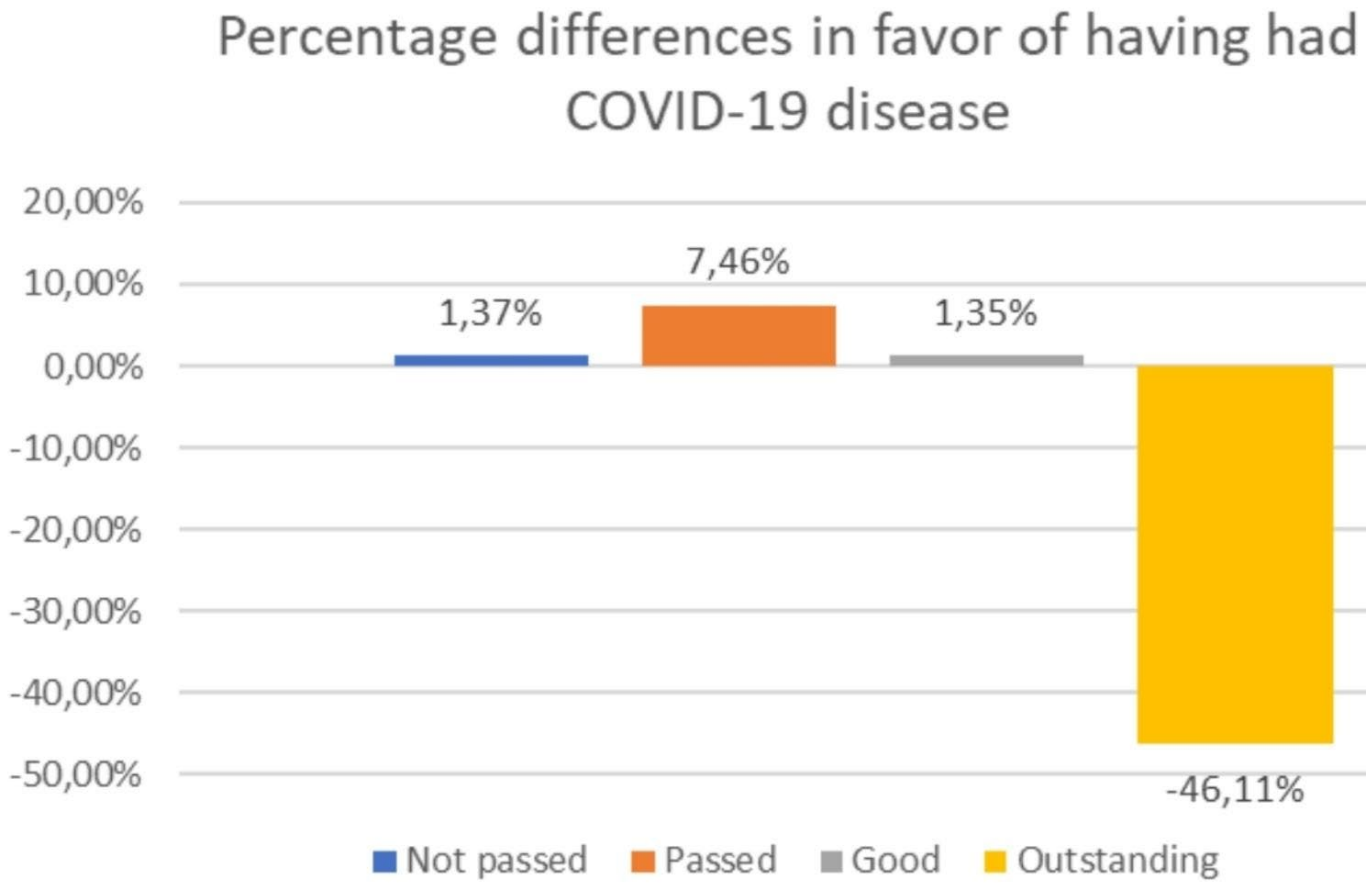
Percentage differences in favor of having had COVID-19 disease

In terms of the mean of academic results and percentages of credits passed, there were significant differences between the two groups, where uninfected students obtained a mean grade of 7.24 (SD 1.36) vs. 7.12 (SD 1.27, p = 0.03) among infected students, and a mean of 93.5% (SD 1.6%) credits passed vs. 93.2% (SD 1–6%, p = 0.050) among infected students. Differences in academic performance classified by four strata are shown in Fig. 2.

After performing the multi-adjusted analyses, it was observed that students with COVID-19 were 46% less likely to have outstanding academic performance with respect to those who didn’t have COVID-19 (Table 2).

**Fig. 3:**
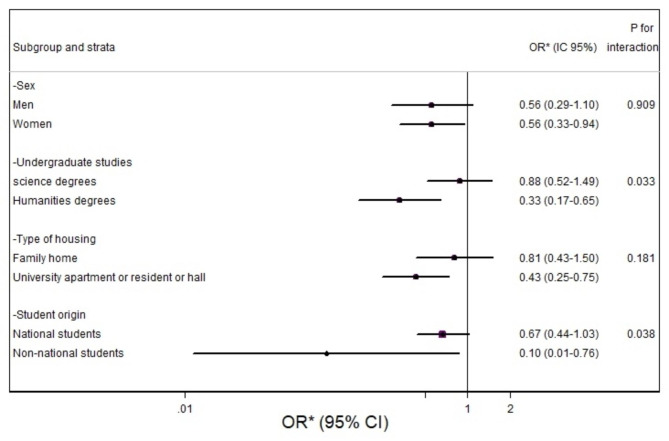
Odds Ratios of students that had COVID-19 relevant characteristics and outstanding academic results. Stratified analysis between students that had COVID-19 and outstanding academic. *Adjusted for sex, age, BMI, smoking status, type of housing, pets during the academic year, region of origin, university campus, year of study, daily hours of socialization

In the stratified analyses, a significant inverse association was found between COVID-19 infection and outstanding academic results in females, humanities students, those living away from their family home, and non-national students (Fig. 3). In addition, a statistically significant multiplicative interaction was found for university undergraduate studies and origin with outstanding academic performance (p-interaction = 0.033 and 0.038), respectively.

**Table 2:** Association between COVID-19 infection and outstanding academic results (infection before any exams)

High score	Non COVID- 19	COVID- 19	p
N	6381	658	
Cases	468	26	
Crude OR	1(ref.)	0.52 (0,35-0.78)	0.001
Sex-, age-adjusted OR	1(ref.)	0.53 (0.35–0.79)	0.002
Multivariable-adjusted OR*	1(ref.)	0.57 (0.38–0.86)	0.008

*Adjusted for sex, age, type of housing, BMI, smoker, academic course, hours of socialization, university campus, pets during the academic year and region of origin

## Discussion

In this prospective cohort study, an association between infection by SARS-CoV-2 and the decrease in academic performance in having outstanding results was found.

This association can be explained by the set symptoms that persistent COVID-19 produces and that negatively impact academic performance. In this known set of several persistent symptoms, some of them lasted for more than 6 months [29]. Persistent fatigue is one of the main sequelae after having COVID-19, with a prevalence of 55% using the Fatigue Severity Scale [29], having a direct impact on physical [23], cognitive performance [24] and labor performance [22]. Headache has also been defined as a post-COVID-19 symptom with a prevalence of 44% [30]. In this sense, it is known that headaches can cause significant disabilities in adolescents and adults by affecting their lives, their academic performance and their relationships with others [20]. In addition, it is known that COVID-19 infection can produce severe impairment of GABA-ergic intracortical circuits in patients who recovered from COVID-19 with various central and peripheral neurological manifestations and who presented fatigue and impairment of executive functions, which causes a decrease in productivity and labor performance [18, 19].

We have observed that two significant factors lower the probability of having outstanding academic results in those students who had COVID-19: studying a humanities degree, with a 67% less than studying sciences degree, and being Non-national students (mostly Latin American), with a 90% less than being Spanish. It is known that COVID-19 generates higher infection rates and causes more severe symptoms in Latin Americans and they present a higher vulnerability to the disease as well as a greater probability of suffering Long COVID effects, which could directly affect their academic and intellectual performance in achieving outstanding academic results.

This result could be explained by the fact that there is a higher proportion of Latin American students studying humanities degrees, 19.21% of the total students of humanities degrees, with regard to 6.82% of Latin American students of the total students studying science degrees. Comparably between both groups of students studying sciences and humanities degrees, there are almost three times as many Latin American students in the humanities degrees group, and as other studies observed, there is a significant difference in how COVID-19 affects Latin American people, being an ethnic group that presented higher positive testing, infection rates and cumulative incidence of the disease [31–33].

The present study has some limitations. First, in this study we did not measure symptoms to verify our hypothesis and, despite this, we have found significant results. According to this hypothesis, if we had measured symptoms and divided our population into three different groups (with persistent COVID, without persistent COVID and uninfected), we could have found results with a greater difference between the magnitudes. A second limitation is that the academic results used are the result of midterm, final, and make-up exams, with some students having passed covid after an evaluation test.

The strengths of the present study include the analysis of almost the entire population of students. In addition, both independent and outcome variables are not self-reported and are measured objectively. In addition, after sensitivity analyses the consistency of the results was maintained.

COVID-19 in the young population is a mild disease without major consequences and death, but still, it may cause long COVID effects that may negatively affect their daily life and academic performance. So, regardless of the fact that they may put other more vulnerable groups at risk, it is interesting to prevent COVID-19 infections in this population group as well.

## Conclusion

In conclusion, COVID-19 in university students is significantly related to not having outstanding results. Future studies should be performed taking into account post-COVID-19 symptoms to confirm our results.

## Data Availability

Data can be requested from Alejandro Fernandez-Montero by e-mail afmontero@unav.es.

## List of abbreviations

BMI: Body mass index
CI: Cumulative incidence
COVID-19: Coronavirus disease 2019
FFP 2/3: Filtering Face Piece 2/3
GABA: Gamma-aminobutyric acid
SARS-CoV-2: Severe acute respiratory syndrome coronavirus 2
OCD: Obsessive compulsive disorder
OR: Odds ratio
PCR: Polymerase chain reaction
PTSD: Post-traumatic stress disorder
STROBE: Strengthening the reporting of observational studies in epidemiology
